# Molecular mechanism on autophagy associated cardiovascular dysfunction in *Drosophila melanogaster*

**DOI:** 10.3389/fcell.2025.1512341

**Published:** 2025-03-03

**Authors:** Wei Zhang, Rong Zhou, Xinjuan Lei, Mofei Wang, Qinchun Duan, Yuanlin Miao, Tingting Zhang, Xinjie Li, Zhang Zutong, Liyang Wang, Odell D. Jones, Mengmeng Xu, Joseph Bryant, Jianjie Ma, Yingli Liu, Xuehong Xu

**Affiliations:** ^1^ Laboratory of Cell Biology, Genetics and Developmental Biology, Shaanxi Normal University College of Life Sciences, Xi’an, China; ^2^ University Laboratory Animal Resources (ULAR), University of Pennsylvania School of Medicine, Philadelphia, PA, United States; ^3^ Department of Pediatrics, Morgan Stanley Children’s Hospital, Columbia University, New York, NY, United States; ^4^ Institute of Human Virology, University of Maryland School of Medicine, Baltimore, MD, United States; ^5^ Division of Surgical Sciences, Department of Surgery, University of Virginia Medical School, Charlottesville, VA, United States; ^6^ Department of Internal Medicine, University Hospital Shaanxi Normal University, Xi’an, China

**Keywords:** *Drosophila melanogaster*, Autophagy, Cardiovascular dysfunction, cardiac laminopathy, mTOR pathway, interference

## Abstract

As a highly conserved cellular process, autophagy has been the focus of extensive research due to its critical role in maintaining cellular homeostasis and its implications in cardiovascular pathogenesis. The decline in muscular function, along with the neuronal system, and increased sensitivity to stress have been recognized in multiple animal models. Autophagic defects in cardiovascular architecture and cellular dysfunction have been linked to both physiological and pathological conditions of the heart in mammals and *Drosophila*. In this review, we systematically analyze the autophagy-associated pathways in the hearts of fruit flies and aim to provide a comprehensive understanding for developing potential treatments for patients and effective strategies for agricultural applications. This analysis elucidates the molecular mechanisms of autophagy in cardiovascular function under both physiological and pathological conditions in *Drosophila*, offering significant insights into the development of cardiovascular diseases. The loss of key autophagy-associated proteins, including the transmembrane protein Atg9 and its partners Atg2 or Atg18, along with DmSestrin, leads to cardiac hypertrophy and structural abnormalities in *Drosophila*, resembling the age-dependent deterioration of cardiac function. Members of the autophagy-related (Atg) gene family, cellular or nuclear skeletal lamins, and the mechanistic or mammalian target of rapamycin (mTOR) signaling pathways are critically influential in heart function in *Drosophila*, with autophagy activation shown to suppress cardiac laminopathy. The mTORC1/C2 complexes, along with axis of Atg2-AMPK/Sirt1/PGC-1α pathway, are essential in the hearts of both mammals and fruit flies, governing cardiac development, growth, maturation, and the maintenance of cardiac homeostasis. The beneficial effects of several interventions that enhance cardiac function, including exercise and cold stress, can influence autophagy-dependent TOR activity of the serine/threonine protein kinase signaling in both mammals and *Drosophila*. Exercise has been shown to increase autophagy when it is deficient and to inhibit it when it is excessive, highlighting the dual role of autophagy in cardiac health. This review evaluates the functional significance of autophagy in the heart, particularly in the context of *Drosophila*, in relation to mTORC-associated autophagy and the axis of Atg2-AMPK/Sirt1/PGC-1α pathways. It systematically contrasts the molecular mechanisms underlying autophagy-related cardiovascular physiological and pathological conditions in both fruit flies and mammals. The evolutionary conservation of autophagy underscores the value of *Drosophila* as a model for understanding broader mechanisms of autophagy across species. This study not only deepens our understanding of autophagy’s role in cardiovascular function but also provides a theoretical foundation for the potential application of autophagy in agricultural pest control.

## 1 Introduction

Autophagy is a vital cellular process responsible for maintaining homeostasis by degrading and recycling damaged cellular components. This process is conserved across species, playing a crucial role in regulating cellular quality control and adaptation to environmental stresses. In both *Drosophila* and mammals, autophagy is activated in response to a variety of stresses, including ischemia and metabolic challenges, to protect tissue integrity and function. Recent studies have shown that autophagy is essential for maintaining cardiac health, with its mechanisms conserved in both species (Santovito et al., 2023). In *Drosophila*, for example, the overexpression of the *Atg2* gene, which is involved in autophagy, enhances cardiac function, and exercise has been found to stimulate autophagic activity, highlighting its importance in heart health (Wen et al., 2023; Zhou et al., 2019; Ghosh et al., 2022). The *Drosophila* homolog of the myokine Irisin, Idit (human FNDC5), regulates both autophagy and exercise performance (Sujkowski et al., 2015; King et al., 2024), further emphasizing the significant role of autophagy in the heart (Cobb et al., 2023; Huang et al., 2022).

Autophagy’s significance is particularly evident in cardiac diseases, including hypertrophy, laminopathies, and age-related cardiac dysfunction. In *Drosophila* models of cardiac laminopathies, for instance, elevated Nrf2 levels inhibit autophagy through mTOR activation, demonstrating the complex relationship between redox signaling and autophagy (Boťanská et al., 2022; Bhide et al., 2018). These findings are echoed in mammalian models, where modulating autophagy has shown therapeutic potential for treating cardiac hypertrophy and age-related dysfunction (Zang et al., 2020a; Ghosh et al., 2020). In particular, the mTOR signaling pathway, a central regulator of autophagy, plays a pivotal role in linking chronic stress and mitochondrial dysfunction to autophagic imbalance in cardiovascular diseases. mTOR inhibition, such as through dietary intake of alpha-ketoglutarate, has been shown to extend lifespan and improve cardiac function in *Drosophila* by modulating autophagy (Su et al., 2019).

Further exploration of autophagy mechanisms in cardiac tissues, particularly through the modulation of mTOR and AMPK pathways in *Drosophila*, provides valuable insights into developing therapeutic strategies for cardiovascular diseases (Walker et al., 2023; Le Dour et al., 2022; Shaw et al., 2022). Investigating these pathways may offer novel approaches to improve heart health and resilience in humans by leveraging conserved mechanisms of autophagy (Sanches-Silva et al., 2020; Georgila et al., 2020; Ren et al., 2021; Xue et al., 2021). In summary, understanding autophagy’s role in cardiac function, particularly through exercise and cold exposure in *Drosophila*, offers valuable insights into improving heart health and resilience in human. Investigating the mTOR and AMPK-mTOR pathway mediated autophagy in *Drosophila* cardiac tissue is crucial for developing therapies for cardiovascular diseases.

## 2 Autophagy and its role in *Drosophila melanogaster*

As the process through which cells degrade and recycle damaged organelles and proteins, autophagy is essential for maintaining cellular health and homeostasis. This cellular process is a key mechanism for the turnover of cellular components in eukaryotic cells, which plays significant roles in growth, development, and responses to environmental stresses. The principal proteins involved in autophagy, initially identified in yeast (*Saccharomyces cerevisiae*), are known as Atg proteins (Tsukada and Ohsumi, 1993). In mammals, three different types of autophagy have been identified, *i.e.*, microautophagy, macroautophagy, and chaperone-mediated autophagy based on their cellular characteristics.

**FIGURE 1:**
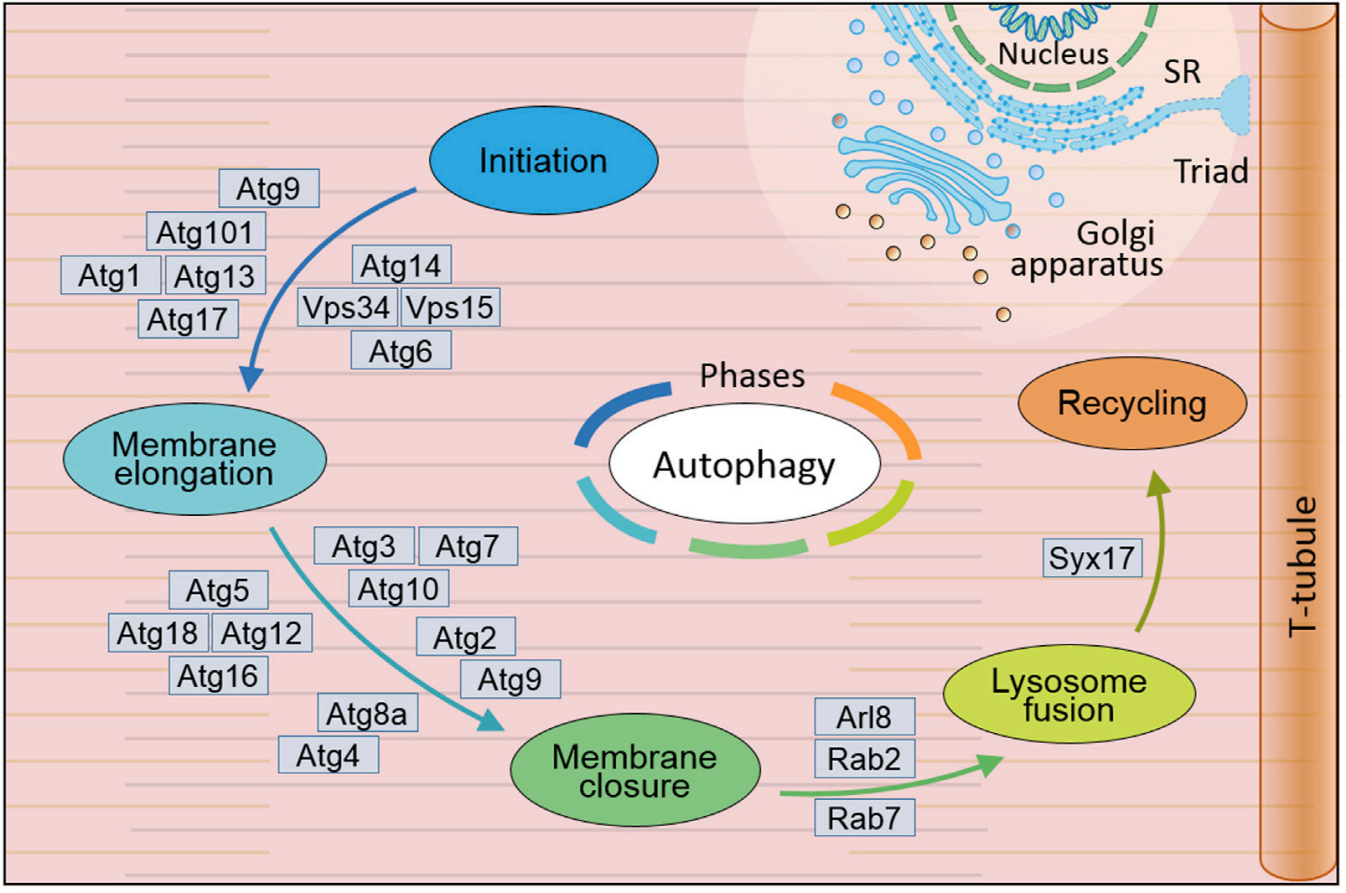
General mechanism of autophagy in *Drosophila melanogaster*. Autophagy occurs through five distinct phases: initiation, during which the autophagic process is triggered; membrane elongation, where the autophagic membrane extends; membrane closure, ensuring the formation of a sealed autophagosome; lysosome fusion, enabling the breakdown of engulfed materials; and recycling, facilitating the reuse of degraded components. This complex autophagy process is orchestrated by the sequential and coordinated action of Atg proteins and their complexes. In *Drosophila*, extensive research has led to the identification of over 20 genes and their coding proteins till now, which have been demonstrated to directly participate in the cellular autophagy. SR, sarcoplasmic reticulum. (Not to scale).

Generally, autophagy works through five phases if cell begin this cellular process, which are initiation, membrane elongation, membrane closure, lysosome fusion and recycling (Figure 1) (Yamamoto et al., 2023). While starvation triggers autophagy initiation, ATG9-associated vesicle associates with ULK1-ATG13/101-FIP200 (fly Atg1-Atg13/101-Atg17) complex and anchors on endoplasmic reticulum (ER) entering second phase membrane elongation. PI3KC3 complex I composed of BECN1 (fly Atg6), VPS15/VPS34 (fly Vps15/Vps34) and NRBF2 along with ATG14 (fly Atg14) is recruited for omegasome formation on the anchored ER locus with multiple ATGs including GABARAP/ATG5/12/16L1 (fly Atg5/8/12/16) and WIPI2 (fly Atg18). Under involvement of ESCORT-III and VPS4, third phase membrane closure is completed accompanied with isolating from ER and forming cellular autophagosome. Mediated with SNAREs and its complex, fourth phase autophagosome accomplishes its tethering fusion with lysosome to degradation and form autolysosome. Further autophagosome enters fifth phase recycling to form recycled ATG9-STX17 (fly Atg9-Syx17) vesicle for next-round autophagic process and cellular reusable protolysosome (Yamamoto et al., 2023). With these four continuously phases, autophagy performs its important role in multiple critical physiological and pathological function processes.

In *Drosophila*, more than 20 genes have been identified to directly associate with cellular autophagy and their regulation in genomic and transcription level. Their encoding proteins involved to its biochemical roles along with other related gene including induction, nucleation and expansion (Demir and Kacew, 2023). Atg1 (human ULK1) with Serine/threonine kinase domain is necessary to trigger autophagic initiation through phosphorylation of Atg13 (human ATG13) on Atg1-Atg13-Atg17 (human ULK1-ATG13-FIP200) complex*.* Interaction between Atg17 (human FIP200) and Atg101 (only in Yeast) can stabilize the complex to grantee the initiation in either mammal or *Drosophila* (Kawamata et al., 2005; Kawamata et al., 2008). As critical factor of ubiquitination, ubiquitin is necessary for cargo-driven assembly of SQSTM1 (fly Ref(2)P) condensate in autophagy initiation and autophagosome formation (Yamamoto et al., 2023). In *Drosophila*, Atg7 (human ATG7), Atg8a (human GABARAP), Atg8b (human GABARAP), Atg10 (human ATG10) and Atg12 (human ATG12) are essential for autophagic initiation by directly involved in the assembly through ubiquitination: Atg7 (human ATG7) as E1-type ligase activating Atg8 (human GABARAP) and Atg12 (human ATG12) enzyme to SQSTM1 (fly Ref(2)P) condensate (Liu J et al., 2024; Turco et al., 2019; Juhász and Neufeld, 2008; Donde et al., 2020); Atg8a (human GABARAP), Atg8b (human GABARAP) and Atg12 (human ATG12) as ubiquitin like protein involving in autophagosome formation (Liu W. et al., 2018; Lőw et al., 2013; Jipa et al., 2021; Xu et al., 2024); (human ATG10) as E2-like enzyme assembly most likely performing its role on autophagy fifth phase recycling (Mizushima et al., 1998; Demir and Kacew, 2023; Damulewicz et al., 2022). Besides Atg8a (human GABARAP), Atg6 (human BECN1), Atg14 (human ATG14) and Atg101 (human ATG101) are necessary autophagosome assembly and/or late endosome-lysosome maturation (Shen et al., 2022; Shravage et al., 2013; Chau et al., 2024; Bánréti et al., 2012; Guo et al., 2019; Hegedűs et al., 2014; Hegedűs et al., 2016). It appears that fruit fly autophagy associated gens is diverse between *Drosophila* fruit flies and mammalians, then we listed in Supplementary Table S1 according to databases of Flybase (https://flybase.org/), NCBI (https://www.ncbi.nlm.nih.gov/gene/), HGNC (https://www.genenames. org/), and MGI (https://www.informatics.jax.org/).

Using *Drosophila* fruit flies combined with complementary DNA (cDNA) analysis, research data have unveiled that more than 289 gene identified to functional corresponding genes, which are related to human diseases including diabetes, autism, cancer, vascular disorder, neuronal degeneration and aging (Yamamoto et al., 2023; Rubin et al., 2000; Santarelli et al., 2023). Currently, many research focuses on molecular mechanism of autophagy and its regulation and revealed function of autophagic flux using *Drosophila* model. Many efforts monitored autophagosome–lysosomal degradation by tracking their movement of cytoplasm, organelles and autophagic-vesicle, and documented time-course of ubiquitinated aggregates during autophagic flux. The data approved that *Atg8* (human *GABARAP*) gene mutations worsen the accumulation of ubiquitinated proteins and over-expressed *Atg8* (human *GABARAP*) avoided this accumulation (Scott et al., 2004; Rusten et al., 2004; Cumming et al., 2008). The above progresses make significant alternation on unveiling autophagy in neurodegenerative diseases induced by proteinopathies (Santarelli et al., 2023). Recently, substantial progresses have been made on autophagy and cardiovascular function using *Drosophila* model, and we summarize the comprehensive detailed on human cardiovascular diseases within following sections.

## 3 Major signaling pathways involved in the regulation of autophagy and heart function in *Drosophila*

As a crucial cellular process involved in degrading and recycling damaged organelles and proteins, autophagy-related (ATG) proteins mediated autophagy plays a significant role in various physiological and pathological conditions. Several signaling pathways regulate autophagy in different contexts. The HIF-1α/BNIP3 signaling pathway induces autophagy and plays a protective role during myocardial ischemia-reperfusion injury (Zhang et al., 2019). Additionally, the mTOR signaling pathway is implicated in regulating autophagy, chronic stress, mitochondrial dysfunction, and senescence, with natural agents like polyphenols potentially modulating this pathway (Sanches-Silva et al., 2020).

### 3.1 Atg proteins regulate autophagy and affect son cardiac function in *Drosophila*

Autophagy is tightly regulated by a network of autophagy-related proteins, with crucial impact on cardiac function in regulating autophagy in *Drosophila*. Murakawa *et al.* found that an extensive tubular autolysosomal network in remodeling muscle is uniquely marked by the autophagic SNARE protein Syntaxin 17 (human STX17). Formation of the network depends on both autophagic flux and degradative cellular process (Murakawa et al., 2020). Based on the data from the Atg-deficient mutants, the efficiency of lysosomal tubulation appeared a co-relationship to phenotypic severity in muscle remodeling, suggesting that Atgs are crucial in regulating autophagy during muscle remodeling. Chang et al. identified TGFβ-INHB (human BMP2)/activin signaling as a novel upstream regulator of mTORC2 to control autophagy and cardiac health during aging. They found that downregulation of TGFβ-INHB/activin activates mTORC2 signaling to regulate cardiac autophagy, and activation of mTORC2 alone promotes autophagic flux and preserves cardiac function with aging in both mouse and fruit fly as well (Chang et al., 2020). Their results approved that Atgs involvement ensure in regulating autophagy and cardiac health in aging *Drosophila*.

An efficient fusion of the autophagosome with the lysosome was found, which may build a critical link for the formation of the tubular autolysosomal network. González-Rodríguez et al. reported that SETD2 (fly Set2) acts as a positive transcriptional regulator of autophagy, primarily regulating the differential expression of protein isoforms encoded by the *ATG14* (fly *Atg14*) gene. They found that, on the condition of nitrogen starvation, SETD2 (fly Set2) promotes the expression of the long ATG14 isoform (ATG14L) but not the short ATG14 isoform (ATG14S), essential for the efficient fusion of the autophagosome with the lysosome (González-Rodríguez et al., 2022). It is worth expecting if this phenomenon would be existent and what a similar function would perform in *Drosophila* especially in heart.

**FIGURE 2:**
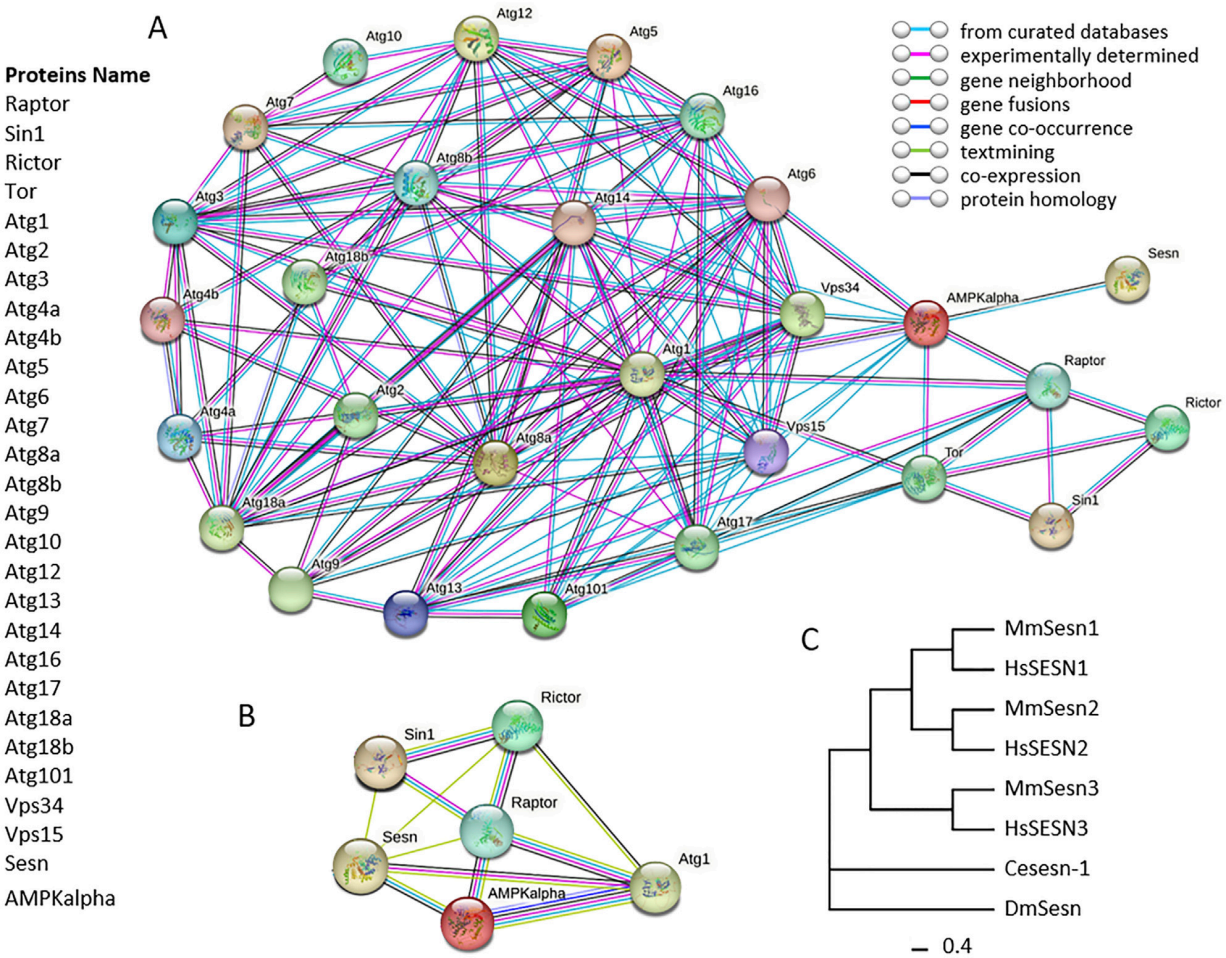
Protein-protein interactions (PPIs) network of autophagy related proteins in *Drosophila melanogaster*. Panel **(A)**, circles in network indicate autophagy related proteins in *D. melanogaster*, and lines in network between circles indicate from curated databases (cyan), experimentally determined (pink) and co-expression (black). Panel **(B)**, circles in network indicate proteins Sesn, Sin1, Rictor, Raptor, AMPKalpha and Atg1. The connecting lines between circles indicate from curated databases (cyan), experimentally determined (pink), gene neighborhood (green), gene fusions (red), gene co-occurrence (blue), textmining (light green), co-expression (black) and protein homology (light blue). The protein-protein interaction networks were constructed using the online STRING server. Panel **(C)**, phylogenetic analysis of Sestrins. The phylogenetic tree was constructed using the Maximum Likelihood (ML) method, incorporating the Sestrin protein sequences of *Drosophila melanogaster* as well as the homologous protein sequences of other model organisms. The tree was generated using the IQ TREE software with the default settings of the software, in which the bootstrap value was set to 1,000. Ce: *Caenorhabditis elegans*, Dm: *Drosophila melanogaster*, Hs: *Homo sapiens*, Mm: *Mus musculus*.

In both mammals and *Drosophila*, Atg1 (human ULK1), composed of three distinct regions—namely, the N-terminal kinase domain, a central region, and the C-terminal MIT domain—is crucial for its interaction with Atg13 (human ATG13) (Toda et al., 2008; Hegedűs et al., 2016). Atg13 (human ATG13) engages with Atg1 via microtubule-interacting and transport domains, facilitated by an extended helix-loop-helix motif. As a binding partner to Atg1, Atg13 is essential for the kinase activity of Atg1 and for the development of the autophagy process (Fujioka et al., 2014; Pan et al., 2019). As the E1-like ubiquitin-activating enzyme, ATG7 (human ATG7) plays a pivotal role in initiating classic autophagy by facilitating the formation and expansion of autophagosome membranes (Liu Y et al., 2024). This protein catalyzes the conjugation of Atg8 (human GABARAP) and Atg12 (human ATG12) to their respective E2 enzymes, Atg3 (human ATG3) and Atg10 (human ATG10) (Tables 1, 2; Figure 2). Remarkably, the interactions between Atg7 (human ATG7) and the E2 core domains of Atg3 (human ATG3) and Atg10 (human ATG10) exhibit strong conservation across different species (Kaiser et al., 2012; Kaiser et al., 2013). These results highlight that comparative studies of autophagic cellular processes in different species should be more comprehensive (Table 1; Supplementary Tables S1–S3). As schematic information and multi-alignment analyses show many critical identities and diversities (Figure 1; Supplementary Figure S1), a more comprehensive study should be performed in *Drosophila*.

**TABLE 1:** Autophagy related proteins domain and functions in *Drosophila melanogaster*.

Gene	Flybase ID	Domain structure and interacting ATG proteins	Functions	References
Atg1	FBgn0260945	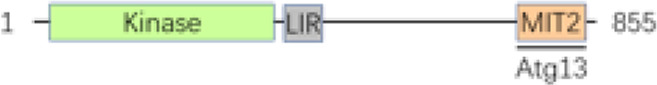	Serine/threonine kinase	Toda et al., 2008; Fujioka et al., 2014; Bierlein et al., 2023; Pai et al., 2023; Srivastav et al., 2024
Atg2	FBgn0044452	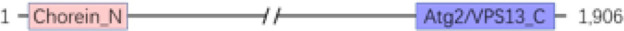	Lipid transporter for autophagosome biogenesis	Shen and Codogno, 2011; Qin et al., 2011; Laczkó-Dobos et al., 2021
Atg3	FBgn0036813	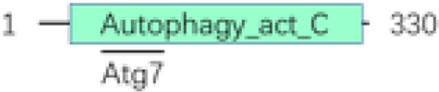	E2-like enzyme, Atg8 ligase activity	Soukup et al., 2016; Ishibashi et al., 2021; Hernandez-Diaz et al., 2022
Atg4a	FBgn0031298	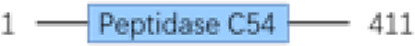	Cysteine-type endopeptidase	Chakraborty et al., 2018; Gerenu et al., 2021
Atg4b	FBgn0038325	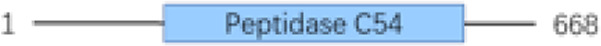	Cysteine-type endopeptidase	Gerenu et al., 2021; Li et al., 2023
Atg5	FBgn0029943	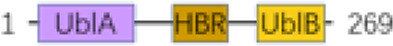	Atg8 ligase activity	Issa et al., 2018; Cui et al., 2020; Insolera et al., 2021; Maruzs et al., 2022
Atg6	FBgn0264325	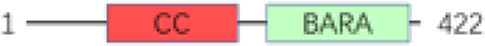	Autophagosome assembly, late endosome-lysosome maturation	Lőrincz et al., 2014; Haghi et al., 2020; Shen et al., 2022
Atg7	FBgn0034366	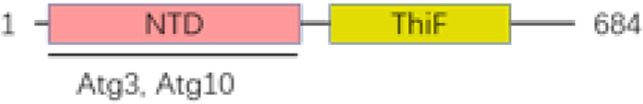	E1-type ligase, Atg8 and Atg12 activator enzyme	Juhász et al., 2008; Pino-Jiménez et al., 2023
Atg8a	FBgn0052672	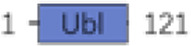	Ubiquitin like protein, autophagosome assembly	Chen et al., 2014; Bali and Shravage, 2017; Jipa et al., 2021; Sujkowski et al., 2022; Zhang et al., 2022
Atg8b	FBgn0038539	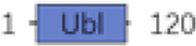	Ubiquitin like protein	Yang et al., 2015; Valko 2019; Perlegos et al., 2024
Atg9	FBgn0034110	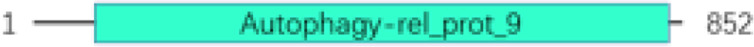	Organization of PAS, response to oxidative stress, JNK activation	Blázquez-Bernal et al., 2021; Yi et al., 2024
Atg10	FBgn0040780	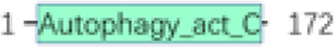	E2-like enzyme, Atg12 transferase activity	Abaquita et al., 2021; Damulewicz et al., 2022; Abaquita et al., 2023
Atg12	FBgn0036255	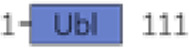	Ubiquitin like protein, Atg8 ligase activity	Pandey et al., 2007; Wang and Zhang, 2019
Atg13	FBgn0261108	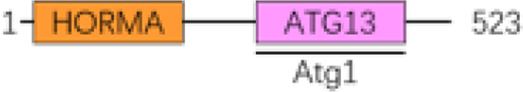	Atg1 regulator activity	Alers et al., 2014; Velentzas and Baehrecke, 2021; Hargitai et al., 2022
Atg14	FBgn0039636	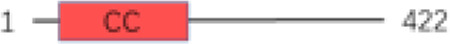	Autophagosome assembly	Khezri et al., 2021; Szabó et al., 2023; Demir and Kacew, 2023
Atg16	FBgn0039705	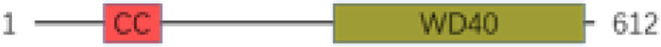	Atg8 ligase activity	Varga et al., 2016; Hargitai et al., 2022; Bhattacharjee et al., 2022
Atg17	FBgn0037363	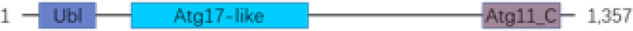	Atg1 regulator activity	Wang and Zhang, 2019; Deng et al., 2022; Hernandez-Diaz et al., 2022; Bierlein et al., 2023
Atg18a	FBgn0035850	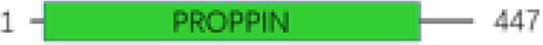	Binds to PI(3,5)P2 and PI3P	Xiao et al., 2018; Shekhar et al., 2023
Atg18b	FBgn0032935	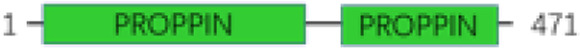	Binds to PI(3,5)P2 and PI3P	DeLeo et al., 2021; Nagata et al., 2022
Atg101	FBgn0030960	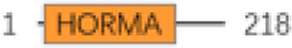	Protein aggregate containing autophagosome formation	Hegedűs et al., 2014; Guo et al., 2019; Jipa et al., 2021
Vps15	FBgn0260935	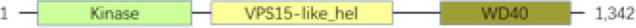	PtdIns 3-kinase	Lindmo et al., 2008; Anding and Baehrecke 2015
Vps34	FBgn0015277	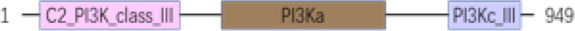	Ser/Thr protein kinase	Juhász et al., 2008; Abe et al., 2009; Csizmadia et al., 2018; Hauswirth et al., 2018
Aduk	FBgn0037679	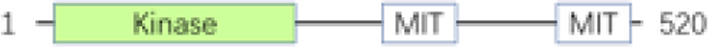	Involved in autophagy and positive regulation of autophagosome assembly	Braden and Neufeld 2016; Jin et al., 2024
Rab2	FBgn0014009	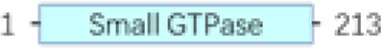	Recruited to the late endosomes, where it controls the fusion of LAMP-containing carriers and lysosomes with the late endosomes	Lőrincz et al., 2017; Lund et al., 2021; Takáts et al., 2021
Rab7	FBgn0015795	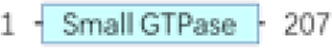	Assists the movement of endocytic compartments, links autophagosomes to dynein/dynactin motors to facilitate movement toward lysosomes	Dinter et al., 2016; Hegedűs et al., 2016; Fujita et al., 2017; Basargekar et al., 2020; Patel et al., 2020; Takáts et al., 2021; Krzystek et al., 2023; Zhou et al., 2023
Sesn*	FBgn0034897	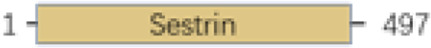	Inhibits the TORC1 signaling pathway	Lee et al., 2010; Kim et al., 2020; Chen et al., 2021; Cobb et al., 2021; Sujkowski and Wessells, 2021; Gu et al., 2022
Syx17	FBgn0035540	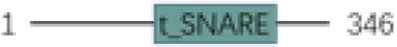	Mediates the fusion of autophagosomes with endosomes and lysosomes	Takáts et al., 2014; Rong et al., 2022
Arl8	FBgn0037551	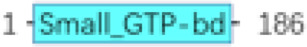	Promotes the proper distribution of lysosomes	Rosa-Ferreira et al., 2018; Boda et al., 2019; Lund et al., 2021

Atg13 engages with Atg1 through a pair of microtubule-interacting and transport domains, facilitated by an extended helix-loop-helix motif. Functioning as a homodimeric E1 enzyme, ATG7 catalyzes the conjugation of ATG8 and ATG12 to their respective E2 enzymes, ATG3 and ATG10.//Break of Atg2 protein sequence. * Suggested autophagy gene in *Drosophila*. Please check out Abbreviations for protein’s full name.

**TABLE 2:** Atg proteins in *Drosophila melanogaster*.

Protein complex	Proteins	Flybase ID	Identity (%)		Phenotype from the loss of function model	References
			Dm/Hs	Dm/Ce		
ATG1 complex	Atg1	FBgn0260945	53.826	66.171	Short lived, oxidative stress response defects, reduced dendritic growth, autophagy defect	Nagy et al., 2014; Ulgherait et al., 2014; Bierlein et al., 2023; Pai et al., 2023; Srivastav et al., 2024
	Atg13	FBgn0261108	43.421	27.778	Short lived, autophagy defect	Alers et al., 2014; Velentzas and Baehrecke, 2021; Hargitai et al., 2022
	Atg17	FBgn0037363	34.043	22.766	Short lived, autophagy defect, reduced locomotor ability	Kim et al., 2015; Wang and Zhang, 2019; Deng et al., 2022; Hernandez-Diaz et al., 2022; Bierlein et al., 2023
	Atg101	FBgn0030960	50.917	33.197	Neurodegeneration, short lived, thicker and shorter midgut, autophagy defect	Guo et al., 2019; Jipa et al., 2021
PID3K complex	Atg6	FBgn0264325	50.115	29.091	Autophagy defect, melanotic mass formation	Lőrincz et al., 2014; Haghi et al., 2020; Shen et al., 2022
	Atg14	FBgn0039636	27.549	--	Autophagy defect. Decreased stem	Khezri et al., 2021; Szabó et al., 2023; Demir and Kacew, 2023
Atg12 and Atg8 conjugation systems	Atg3	FBgn0036813	62.84	57.732	Short lived, autophagy defect	Soukup et al., 2016; Ishibashi et al., 2021; Hernandez-Diaz et al., 2022
	Atg4a	FBgn0031298	41.287	36.32	Accumulation of p62 positive	Chakraborty et al., 2018; Gerenu et al., 2021
	Atg4b	FBgn0038325	33.994	33.333	-	Gerenu et al., 2021; Li et al., 2023
	Atg5	FBgn0029943	48.339	30.741	Memory defect, autophagy defect, ataxia, reduced dendritic growth, impaired immune function	Issa et al., 2018; Cui et al., 2020; Insolera et al., 2021; Maruzs et al., 2022
	Atg7	FBgn0034366	40.741	36.031	Short lived, memory defect, impaired immune function, autophagy defect	Juhász et al., 2008; Pino-Jiménez et al., 2023
	Atg8a	FBgn0052672	91.453	85.345	Reduced dendritic growth, autophagy defect	Chen et al., 2014; Bali and Shravage, 2017; Jipa et al., 2021; Sujkowski et al., 2022; Zhang et al., 2022
	Atg8b	FBgn0038539	91.453	85.345	Male sterility	Yang et al., 2015; Valko, 2019; Perlegos et al., 2024
	Atg10	FBgn0040780	23.853	26.506	-	Abaquita et al., 2021; Damulewicz et al., 2022; Abaquita et al., 2023
	Atg12	FBgn0036255	27.5	27.632	Reduced immune function, autophagy defect	Pandey et al., 2007; Wang and Zhang, 2019
	Atg16	FBgn0039705	41.23	35.762	Defective immune responses, inflammation, thicker and shorter midgut, short lived, autophagy defect	Hargitai et al., 2022; Bhattacharjee et al., 2022
Atg2–PROPPINs	Atg2	FBgn0044452	28.917	30.657	Short lived, decreased cell death, reduced dendritic growth, autophagy defect, reduced locomotor ability, decreased heart function	Shen and Codogno, 2011; Qin et al., 2011; Xu et al., 2019; Laczkó-Dobos et al., 2021; Wang et al., 2023
	Atg18a	FBgn0035850	59.043	39.944	Short lived, reduced dendritic growth, autophagy defect, reduced locomotor ability	Xiao et al., 2018; Xu et al., 2019; Bierlein et al., 2023; Shekhar et al., 2023
	Atg18b	FBgn0032935	59.043	39.944	-	DeLeo et al., 2021; Nagata et al., 2022
Atg9 vesicle	Atg9	FBgn0034110	40.625	35.762	Short lived, thicker and shorter midgut, memory defect, autophagy defect, reduced locomotor ability	Xu et al., 2019; Blázquez-Bernal et al., 2021; Yi et al., 2024

AA, amino acids; PI, isoelectric point; *Dm*, *Drosophila melanogaster*; *Hs*, *Homo sapiens*; *Ce*, *Caenorhabditis elegans;* “-”, loss of function has not been carried out yet in fruit flies; “--”, no homologous protein in *C. elegans*.

Nishimura et al. highlighted the emerging roles of Atg proteins and membrane lipids in autophagosome formation in mouse model. They discussed the ability of Atg proteins to interact directly with membranes, transfer lipids between membranes, regulate lipid metabolism, and the role of various membrane lipids in autophagosome formation (Nishimura and Tooze, 2020). Peng et al. identified critical Atgs, specifically Atg2 (human ATG2), Atg9 (human ATG9), and Atg18 (human WIPI2), as integral for maintaining mitochondrial integrity and cardiac function. The targeted knockdown of Atg2, Atg9, or Atg18 in the heart and indirect flight muscles of *Drosophila* led to a shortened lifespan and a decline in locomotive function, underscoring the importance of the Atg2-Atg18 (human ATG2-WIPI2) or Atg2-Atg9 (human ATG2-ATG9) autophagy complex in preserving mitochondrial health and regulating heart and muscle functions (Xu et al., 2019).

These findings approved that Atgs are involved in the formation of autolysosomal networks, regulation of autophagic flux, and the fusion of autophagosomes with lysosomes, highlighting their importance in maintaining cellular homeostasis and cardiac health in *Drosophila* as well.

### 3.2 Axis of Atg2-AMPK/Sirt1/PGC-1α (human ATG2-AMPK/SIRT1/PGC-1α) pathway postpones age-related deteriorations of skeletal and cardiac muscle in *Drosophila*

Muscular tissues, including skeletal muscle for wing movement and cardiac muscle for circulation in fruit flies, have long been recognized for their autophagy functions. As a critical pathway, autophagy plays a role in maintaining muscle performance and metabolism, which are closely related to aging, including cardiac muscle senescence (Aparicio et al., 2019; Rodney et al., 2016; Fujita et al., 2017). By maintaining muscle mass and myofibril function, autophagy performs its proteolytic role by managing the accumulation of misfolded proteins and dysfunctional mitochondria (Rodney et al., 2016; Terman et al., 2010; Miyamoto, 2019; Hood et al., 2019). Increasing autophagy significantly improves heart function against aging through regulation of the Atg-AMPK pathway axis, which was recently demonstrated in *Drosophila melanogaster*.

When *Atg2* (human *ATG2*) gene was overexpressed in skeletal muscle and heart function of fruit fly, the specific expression significant promoted activation of the AMPK/Sirt1/PGC-1α (human AMPK/SIRT1/PGC-1α) pathway and eventually declined the aging process in male combined with loss of function approach via muscle *Atg2* (human *ATG2*) knockdown (Wang et al., 2023; Kayashima et al., 2017; Fujita et al., 2017). Based on climbing characteristics against gravity, the *Drosophila* fruit flies were trained endurance exercise. Climbing index were used to measure as motor capabilities of the endurance and speed recognition. Using real-time quantitative PCR analysis, the mRNA expression of *Atg2* (human *ATG2*), *Atg8a* (human *GABARAP*), *AMPK* (human *AMPK*), *Sirt1* (human *SIRT1*) and *PGC-1α* (human *PGC-1α*) were inspected. The research conclude that the muscle specific Atg2 overexpression can improve the motor ability via activating AMPK/Sirt1/PGC-1α pathway, and enhancing antioxidant capacity of muscle. This phenomena was confirmed by Atg2 muscle specific knockdown, which reduced the motor capacity through inhibiting AMPK/Sirt1/PGC-1α pathway along with declining muscle antioxidant capacity (Wang et al., 2023).

Furthermore, using M-mode cardiogram inspection characterized with systolic time, systolic diameter, diastolic diameter, cardiac cycle and ejection fraction, the research approved that the muscle *Atg2* (human *ATG2*) expression and endurance exercise effected on heart function of fruit flies. This functional improvements resulted in delaying muscle aging, and significant improved motor capabilities along with prolonged *Drosophila* lifespan (Wang et al., 2023). These effects on heart function evidently linking to AMPK/Sirt1/PGC-1α (human AMPK/SIRT1/PGC-1α) pathway demonstrated that endogenous autophagy Atg2 (human ATG2) plays its role through the AMPK through axis of Atg2- AMPK in *D. melanogaster,* which provides comprehensive understanding of complex effects of autophagy in both mammal and *Drosophila*, as described in previous publications (Aparicio et al., 2019; Terman et al., 2010; Kayashima et al., 2017; Miyamoto, 2019).

### 3.3 mTORC2 associated autophagy against heart senescence and aging in *Drosophila*

The mTOR signaling pathway is associated with various human diseases, including diabetes, cancer, epilepsy, and liver diseases (Konopka et al., 2023; Quarles et al., 2020; Sanches-Silva et al., 2020). Recently many works approved that autophagy tightly associated with this pathway (Sciarretta et al., 2012; Wohlgemuth et al., 2007; Dai et al., 2014; Linton et al., 2015; Miyamoto, 2019; Quarles et al., 2020). It is critical to understanding how autophagy is regulated under different stress conditions and the role of the mTOR signaling pathway, which would be important to apprehend autophagic regulation and its affective role on cardiac function in *Drosophila*.

Using the UAS-Gal4 system to drive the RNAi specific against genes of interest involved in the research, a study showed that when they specifically knocked down (human INHBA) (TGFβ-INHB/activin-like ligand) in the heart, cardiac senescence was significantly slowed down. Daw negatively regulates autophagic flux in *Drosophila* hearts, preserving autophagic activity against cardiac aging, as measured in semi-intact *Drosophila* adult fly hearts using M-modes and cardiac parameters generated with a Matlab-based application (Chang et al., 2020). Remarkably, they demonstrated that Atg1-mediated autophagic activity, determined through lysosomal CtsB1 (human CTSB) activities examined with the Magic Red Cathepsin-B Assay, is critically required for daw-regulated cardiac aging. By targeting mTOR, they showed that this mTOR-Atg1-mediated autophagic activity is primarily achieved via mTORC2-rictor, but not mTORC1, as a negative regulator (Chang et al., 2020). Their data demonstrated that, through the Atg1-mTORC2 complex of Atg1-mediated autophagic activity, cardiac-specific reduction of INHB/activin-daw and rictor (human RICTOR) overexpression significantly prolonged lifespan along with healthspan of the heart in *Drosophila*.

Since mTOR stands for the mammalian target of rapamycin, studying the characteristics of rapamycin-treated fruit flies can be particularly insightful for understanding the mTOR pathway and its function in *Drosophila*. Recently, Regan and Partridge highlighted a significant finding in rapamycin-treated flies: In response to rapamycin, Atg5 (human ATG5) promoted autophagy, playing a positive role specifically in female *Drosophila*, but not in males, by regulating intestinal health and lifespan (Regan et al., 2022). Interestingly, their data revealed that rapamycin treatment reduced p62/SQSTM1 (fly Ref (2) P) levels, affecting not only intestinal epithelial cells but also critical metabolic tissues and organs, including the liver, brown adipose tissue, muscle, heart, kidney, jejunum, and colon (Regan et al., 2022). This finding suggests that rapamycin-induced *Atg5*-autophagy may exert a protective function on both cardiac and intestinal health, along with lifespan extension.

Moreover, the study by Wang et al. demonstrates the role of endonuclease G (ENDOG) (fly EndoG) in promoting autophagy by suppressing the mTOR signaling pathway (Wang et al., 2021). The findings suggest that ENDOG (fly EndoG), released from mitochondria, promotes autophagy during starvation by inhibiting the mTOR pathway, and this mechanism is evolutionarily conserved across species, including *Drosophila* (Yin et al., 2021). Since mitochondria are abundantly distributed and perform critical function in the heart of both mammals and fruit flies, the current understanding of the autophagy-mTOR signaling axis, especially the relationship between post-translational modifications and their role in regulating autophagy in cardiomyocytes, could serve as an effective target for pharmaceutical development.

### 3.4 DmSestrin associated mTOR pathway could be critical for autophagy in *Drosophila*

As a highly conserved stress-responsive protein, SESN2 (fly Sesn) can activate adenosine monophosphate-activated protein kinase (AMPK) and inhibit the mechanistic target of rapamycin complex 1 (mTORC1) to maintain cellular homeostasis by influencing autophagy, including macroautophagy, mitophagy, ER stress, and apoptosis, along with redox regulation, protein synthesis, and inflammation (Lu et al., 2023). In liver diseases such as non-alcoholic fatty liver disease (NAFLD), SESN2 (fly Sesn) slows disease progression by balancing glycolipid metabolism through macroautophagy (lipophagy) and delays the onset and progression of fibrogenesis (Lu et al., 2023). In the mammalian and human heart, both mTORC1 and mTORC2 complexes of the mTOR signaling pathway play critical roles in cardiac physiology and the pathology of cardiovascular diseases, including cardiac fibrosis and inflammation (Lu et al., 2023; Sciarretta et al., 2022; Sciarretta et al., 2018; Gao G. et al., 2020). SESN2 (fly Sesn) and its regulation are also closely associated with cardiovascular diseases, making it a potential therapeutic target (Gao A et al., 2020).

In *Drosophila*, using the fruit fly *UAS-GAL4* system compared to mouse models and 2 cell lines (human embryonic kidney (HEK293) and mouse embryonic fibroblast (MEF)), Kim and Lee revealed that Sesn or DmSestrin (human SESN2) overexpression downregulated mTORC1 activity in AMPK-null MEFs, but directly interacted with GATOR2, not GATOR1, using tandem affinity purification (TAP) coupled with mass spectrometry (MS) and co-immunoprecipitation assays (Kim et al., 2015). Overexpressed Sesn or DmSestrin (human SESN2) enhanced GATOR1 activity toward RagB, leading to mTORC1 inhibition, accompanied by subcellular relocalization of mTOR, which no longer localized with Rag GTPases on the lysosomal membrane (Kim et al., 2015). Similarly, *Drosophila* Sesn or DmSestrin, as the only homologous gene of Sestrin family in flies, in regulates cell growth through GATOR complex modulation, suggesting an identical mechanism in mammals. This was confirmed by a DmSestrin loss-of-function *Drosophila* model, which accurately corrected autophagy defects with GATOR2 downregulation (Kim et al., 2015). Among *Drosophila* ATGs, Vps34 (human VPS34) and Vps15 (human VPS15), Sesn (human SESN2) interacts with AMPK, confirmed through PPI analysis using databases, experiments, and co-expression (Figure 2, Panel A). Additional PPI analysis shows that Sesn (human SESN2) can interact with AMPK (via co-expression, curated databases, and text mining), Sin1 (via text mining), and Rictor (via text mining)/Raptor-TOR (via text mining) within the PPI network of AMPK and Raptor/Rictor-mTOR (Figure 2, Panel B and C). We propose that Sesn should be considered an autophagy gene in *D. melanogaster*.

Based on the above discussion, it is reasonable to conclude that Sesn (human SESN2) -triggered autophagy is important in *Drosophila* for maintaining cardiovascular health, sharing molecular mechanisms with mammals. We speculate that a deeper understanding of *Drosophila* autophagy may reveal whether Sesn (human SESN2) phosphorylation is involved in the autophagic degradation of mitochondria damaged by various stressors (Piochi et al., 2021; Xu et al., 2019), which could provide additional support for developing more effective therapeutic strategies.

## 4 RAS and RAF associated autophagy in cardiac hypertrophy of *Drosophila*

Cardiac hypertrophy, defined by an increase in cardiomyocyte size, serves as an adaptive response to stress but, if uncontrolled, can progress to heart failure, posing significant health risks (Mönck et al., 2017). In the *Drosophila* model of cardiac hypertrophy, research by Wolf’s group has provided valuable insights into the molecular mechanisms driving this condition, particularly focusing on the role of receptor tyrosine kinase (RTK) (human HRAS) signaling pathways associated to the RAS/RAF cascade. The RAS and RAF signaling pathways, key regulators of cell proliferation and survival, have been implicated in the development of cardiac pathologies (Yu et al., 2013). In this model, activated *EGFR* ^*A887T*^, *Ras85D* ^*V12*^, and *Raf* (human *BRAF*) transgenes induce phenotypes similar to those seen in hypertrophic cardiac remodeling, including increased cardiomyocyte ploidy and heart chamber abnormalities (Yu et al., 2013). The RAS/RAF pathway’s role in cardiac hypertrophy has prompted further exploration of its potential as a therapeutic target, particularly in obesity-related heart disease, where impaired autophagy exacerbates structural and functional cardiac changes (Castañeda et al., 2019). These findings highlight the importance of autophagy in modulating the effects of RAS/RAF activation in cardiac hypertrophy.

To investigate cardiac hypertrophy in *Drosophila*, Wolf’s group studied transgenic fruit flies expressing activated *EGFR* ^*A887T*^, *Ras85D* ^*V12*^, and *Raf* (human *BRAF*), using confocal microscopy and histological analysis of heart structure, along with quantification of cardiomyocyte ploidy, heart rate, and rhythm (Yu et al., 2013). Their data revealed that adult flies with activated *EGFR* ^*A887T*^ or *Ras85D* ^*V12*^ showed significantly decreased eclosion frequencies and poor survival. Confocal microscopy revealed that these transgenic heart tubes exhibited smaller heart chambers, increased heart wall thickness, abnormal cardiac morphology, and myofiber disarray (Yu et al., 2013). Expression of activated *Raf* induced an increase in cardiomyocyte ploidy, along with cardiac hypertrophy, similar to that caused by overexpressed *Ras* (Yu et al., 2013). Both Ras signaling and Raf are well-studied pathways closely associated with cardiac pathologies, and many therapeutic targets have been developed for clinical treatment (Ramos-Kuri et al., 2021). Recently, autophagy has been recognized linking to cardiovascular disease and aging in part due to its close relationship with the Ras pathway (Ramos-Kuri et al., 2021; Koutouroushis and Sarkar, 2021; Miyamoto, 2019). Additionally, as a key node in the RAS/RAF/MAPK pathway, RAF family protein kinases are deeply involved in controlling multiple cellular processes, including proliferation, differentiation, and survival, in response to growth factor receptor activation on various cells, including skeletal muscle cells, cardiomyocytes, and metastatic tumor cells (Jeon H et al., 2024). RAF kinases are now being used for both mechanistic and clinical studies as potential therapeutic targets (Bahar et al., 2023). We speculate that Raf-mediated cardiac hypertrophy in *Drosophila* could generate more comprehensive information on hypertrophy using this low-cost and fast animal model.

In the context of cardiac hypertrophy, the interaction between the RAS/RAF signaling pathways and autophagy has emerged as a critical area of investigation. Recent studies in *Drosophila* have demonstrated that activation of the RAS/RAF cascade, through transgenic expression of *EGFR* ^*A887T*^, *Ras85D* ^*V12*^, and *Raf* (human *BRAF*), induces phenotypes similar to hypertrophic remodeling, including increased cardiomyocyte ploidy and structural heart abnormalities (Ramos-Kuri et al., 2021; Koutouroushis and Sarkar, 2021; Miyamoto, 2019; Xu et al., 2019). In *Drosophila*, targeted knockdown of autophagy-related genes such as Atg2, Atg9, or Atg18 in cardiac and flight muscles resulted in shortened lifespan and accelerated age-related cardiac deterioration, characterized by increased heart tube wall thickness and structural abnormalities (Xu et al., 2019). Using transmission electron microscopy and the Mef2-GAL4-MitoTimer mitochondrial strategy, they revealed elongated mitochondria and a reduction in autophagosomes containing mitochondria in the heart tubes, while indirect flight muscles showed heightened mitochondrial fragmentation and reduced mitochondrial density (Xu et al., 2019). These findings underscore the importance of autophagy in maintaining mitochondrial health and preventing cardiac dysfunction. Additionally, Martinelli et al. (2021) identified a role for galanin in regulating cardiac autophagy and reducing apoptosis in hypertrophied hearts through the FOXO (Human FOXO) pathway, thereby preserving mitochondrial integrity and offering new insights into cell survival mechanisms during cardiac remodeling (Martinelli et al., 2021). These studies suggest that enhancing autophagy, particularly the Atg2-Atg18/Atg9 complex activity, may offer therapeutic potential for ameliorating hypertrophic cardiomyopathy and related disorders.

## 5 Cardiac laminopathy and autophagy in *Drosophila*

Mutations in the human LMNA (fly *LamC*) gene cause laminopathies, including cardiomyopathies characterized by arrhythmias and heart failure (Bhide et al., 2018). *Drosophila* models of skeletal muscle laminopathies have been used to investigate the pathological effects of mutant lamins and identify potential therapeutic targets (Chandran et al., 2019). Autophagy plays a key role in mitigating cardiac laminopathy, with age-dependent autophagy deficiency linked to Nrf2-mediated dysfunction (Zang et al., 2020a). Activating autophagy, along with modulating muscle redox and protein aggregation, has been shown to rescue cardiac function and extend lifespan (Coombs et al., 2021; Villanueva et al., 2019). In *Drosophila* models, elevated Nrf2 levels inhibit autophagy via mTOR activation, but blocking Nrf2 and enhancing autophagy can alleviate age-related cardiac dysfunction and improve lifespan (Boťanská et al., 2022; Villanueva et al., 2019). These findings underscore the therapeutic potential of autophagy activation in cardiac laminopathy.

### 5.1 Increasing autophagy suppresses laminopathy-induced age-dependent cardiac dysfunction

Increasing autophagy and blocking Nrf2 have been shown to suppress laminopathy-induced age-dependent cardiac dysfunction and shortened lifespan in *Drosophila* (Bhide et al., 2018). This indicates that autophagy plays a crucial role in mitigating the effects of laminopathy on cardiac function. Additionally, time-restricted feeding has been found to restore muscle function in *Drosophila*, further supporting the potential of autophagy modulation in addressing age-related cardiac dysfunction (Bhide et al., 2018). The interplay between autophagy and redox signaling has also been implicated in regulating cardiac function, underscoring the importance of understanding the molecular and mechanobiological pathways related to autophagy in the context of laminopathy-induced cardiac dysfunction (Bhide et al., 2018; Boťanská et al., 2022; Coombs et al., 2021).

Using *Drosophila* as a model organism, Bhide et al. introduced human disease-causing mutations into the *LamC* gene (the mammalian *LMNA* homologue) and expressed the mutant LamC protein in cardiomyocytes. This manipulation led to progressive cardiac dysfunction, disruption of adipose tissue homeostasis, and a shortened lifespan in adult flies. The findings suggest that an imbalance in autophagic flux and activation of the Nrf2/Keap1 pathway contribute to the pathogenesis of cardiac laminopathies. Concurrent enhancement of autophagy and inhibition of the Nrf2/Keap1 pathway may represent a potential therapeutic approach for these conditions (Bhide et al., 2018). Additionally, the Keap1-Nrf2 system has been identified as a mediator between oxidative stress and cardiac dysfunction, suggesting that targeting Nrf2 and autophagy may hold promise for addressing laminopathy-induced cardiac dysfunction (Bhide et al., 2024; Yu and Xiao, 2021; Zang et al., 2020b). These findings highlight the potential of autophagy modulation as a therapeutic strategy for mitigating age-dependent cardiac dysfunction in the context of laminopathy.

### 5.2 Mutant LamC causes abnormal autophagic defects and mitochondrial dysmorphology

Using the short lifespan and conserved cardiac proteome of *Drosophila*, Kirkland et al. found that cardiomyocytes exhibit a progressive loss of LamC (human LMNA) with age, accompanied by a decrease in nuclear size and an increase in nuclear stiffness. Premature genetic reduction of LamC mirrors the effects of aging on the nucleus, subsequently reducing cardiac contractility and sarcomere organization (Kirkland et al., 2023). The study of mutant LamC in *Drosophila* has revealed various abnormalities in cellular processes. LamC K521W and R564P mutations have been shown to cause nuclear and cytoplasmic abnormalities in larval fat body tissue, indicating defects in cellular structure and function (Walker et al., 2023).

Additionally, these mutations have been associated with held-up wings, indicative of myofibrillar defects and mitochondrial damage in *Drosophila* (Kucherenko et al., 2010). These findings suggest that mutant LamC can lead to abnormal autophagic defects and mitochondrial dysmorphology in *Drosophila*, highlighting the importance of understanding the impact of these mutations on cellular processes (Walker et al., 2023). Further research may help identify potential therapeutic targets for addressing the cellular defects caused by mutant lamins. At the cellular level in cardiac cells, the mutant LamC protein accumulated in the cytoplasm, leading to the activation of the Nrf2/Keap1 redox signaling pathway. The mitochondria displayed abnormal morphology, and there was an upregulation of the autophagy cargo receptor Ref(2)P/p62 (Bhide et al., 2018).

### 5.3 Association of autophagy and nuclear remodeling and myogenic transcription factors in cardiac remodeling in mouse, *Drosophila* and non-human primates

Despite physiological differences between tubular and chambered hearts, there is noteworthy overlap in the cardiac proteomes of *Drosophila*, mice, and the non-human primate Rhesus macaque (Kirkland et al., 2023), which is certain that the cardiac proteome tightly related to autophagy via NRF2 signaling pathway (NRF2, a nuclear factor erythroid 2-related factor 2). Kirkland et al. found that senescence is a Lamin-dependent biological process that induces cardiac dysfunction characterized by dysregulation of cardiac transcriptional programs in *Drosophila* and mammalian animals, including mice and macaques, designating similarities with the human heart. In aging *Drosophila*, cardiac senescence is characterized by reduced LamC, decreased nuclear size, and increased nuclear stiffness. Interfering with LamC RNA expression using UAS-LamC-RNAi resulted in downregulation of cardiac transcription factors accompanied by declined heart function. And meanwhile, enhancing autophagy by blocking the Nrf2 signaling pathway moderated cardiac dysfunction and extended the lifespan of *Drosophila*, a phenomenon also observed in mice and macaques (Kirkland et al., 2023). In their study, they demonstrated that the enhanced autophagy and MTF loss are strongly associated with the reduction of Lamin C through blocking the NRF2 signaling pathway. With aging, the occurrence of nuclear remodeling and myogenic transcription factor (MTF) loss was accompanied by alterations in cellular structures, such as those within the cell nucleus, where the integrity of the cardiac cell nucleus is necessary for maintaining cardiac functions. This association can alleviate cardiac dysfunction in organisms experiencing senescence (Kirkland et al., 2023). Aguiari et al. demonstrated that silencing COUP-TFII restored the myogenic potential of TRα1PV satellite cells, leading to enhanced myoblast proliferation and myotube differentiation in a mouse model (Aguiari et al., 2021). Their data may link to the results on myogenic characteristics observed by Kirkland et al., where time-restricted feeding induced cardiac dysfunction and decreased the food-limitation triggered autophagy through dysregulation of cardiac transcriptional programs in *Drosophila*, mice, and macaques, highlighting the importance of nuclear integrity in cardiac health across these organisms (Kirkland et al., 2023).

As the transcription factor that regulates multiple cellular defenses against toxic and oxidative insults in mammals, autophagy associated NRF2 (Kirkland et al., 2023) carries out its function by promoting gene expression in response to oxidative stress, energy metabolism, and inflammation in both physiological and pathological states (He et al., 2020; Saha et al., 2020; Bendavit et al., 2016). As a pleiotropic transcription factor, many of its cellular functions are involved in autophagy, making it a critical player in cardiac pathophysiology closely associated with mitochondrial responses. Rabinovich-Nikitin et al. identified a circadian CLOCK-mitochondrial interactome that regulates mitochondrial autophagy and cell survival in cardiac myocytes during ischemic stress (Rabinovich-Nikitin et al., 2021). Zang et al. reported that autophagy inhibition allowed NRF2 to exacerbate the progression of diabetic cardiomyopathy in a mouse model, suggesting a complex role for NRF2 in cardiac health (Zang et al., 2020b).

Furthermore, Bendavit et al. claimed that Nrf2 is a master transcription factor that regulates a wide variety of cellular proteins, as mentioned above, through its binding domain on gene promoter regions. Increasing cellular autophagy associated NRF2 promotes the transcriptional activation of the PI3K signaling pathway for mTOR transcription (Bendavit et al., 2016). Pan et al. found that cardiomyocytic FoxP3 was involved in Parkin-mediated mitophagy during cardiac remodeling, emphasizing the regulatory role of transcription factors in cardiac health (Pan et al., 2022). Fang et al. showed that soluble epoxide hydrolase inhibition protected against diabetic cardiomyopathy by inducing autophagy and reducing apoptosis through Nrf2 upregulation and transcriptional activation (Fang et al., 2022).

A translation repressor protein was approved linking to autophagy through the mTOR signaling pathway. In *Drosophila*, Zid et al. published data revealing that food stress significantly increased nuclear-encoded mitochondrial genes, Complex I and IV of the electron respiratory chain, while diminishing lifespan extension, as assessed with the nuclear protein Lamin A and cytoplasmic Akt (human AKT) (Zid et al., 2009). As a translation repressor protein and a well-known autonomous target of the mTOR signaling pathway (Qin et al., 2016), the eukaryotic initiation factor eIF4E (human EIF4E) binding protein, 4E-BP (human EIF4EBP1), is essential for appropriate cardiac performance by tightly regulating Ca^2+^ handling associated with Ca^2+^ ATPase and SERCA (human ATP2A) in the *Drosophila* heart (Santalla et al., 2022). In response to overexpression of the autophagy inducer kinase Atg1, FOXO, and 4E-BP (human EIF4EBP1) CA in fruit fly muscles, their data demonstrated that the 4E-BP-Pten/FOXO (human EIF4EBP1-PTEN/FOXO) signaling axis in *Drosophila* muscles is critical for preserving proteostasis during aging through regulation of the autophagic lysosome system, which is marked by extending the lifespan of fruit flies (Demontis and Perrimon, 2010).

It was confirmed early that Dm4eBP associated DmTOR and dFoxo are key regulators of cardiac aging, responding to changes in nutritional and environmental conditions to control processes that preserve heart function in *Drosophila* (Wessells et al.). DmTOR, which regulates growth and metabolism, and dFoxo, a stress-responsive transcription factor, both influence the rate of age-related cardiac decline by modulating cellular stress responses and protein damage accumulation in flies (Wessells et al., 2009; Luong et al., 2006). Notably, d4eBP, a downstream effector of both DmTOR and dFoxo, elevates d4eBP levels in the myocardium enhancing stress resistance, maintains myogenic rhythm, and prevent functional decline (Wessells et al., 2009). Furthermore, Demontis and Perrimon, 2010 show that FOXO and 4E-BP signaling maintain proteostasis via autophagy (Demontis and Perrimon, 2010). Inhibition of DmTOR activates autophagy, counteracting the accumulation of damaged proteins associated with aging (Nyfeler et al., 2011). Thus, the coordinated activity of DmTOR, DmFoxo, and Dm4eBP promotes autophagy in the heart, slowing the cardiac functional decline typical of aging (Wessells et al., 2009; Luong et al., 2006). Schmid et al. approved that accumulation of F-actin even associated with the d4eBP-dTOR-dFoxo axis on brain aging phenotypes and prolong healthspan (Schmid et al., 2024).

Autophagy associated TOR and FOXO associated nuclear transcription factors along with lamins located in the nuclear cortex, are functionally and structurally fundamental for nuclear remodeling, which is genetically and physiologically necessary for the maintenance of metabolism and senescence in the cardiac and skeletal muscles of both mammals and fruit flies.

## 6 Exercise improves cardiac function and againgst aging through autophagy in *Drosophila*

Recently, extensive evidence supports the beneficial role of exercise or physical activity in cardiovascular health, partially through its regulation of autophagy. The relationship between exercise and autophagy is complex and bidirectional. In conditions where autophagy is either deficient or excessive, exercise training restores normal autophagic activity, thereby potentially delaying the progression of cardiovascular diseases associated with dysfunctional autophagy (Wang et al., 2020). Specifically, exercise induces autophagy in the myocardium, contributing to beneficial effects on cardiac function against age-related muscle deteriorations (Wang et al., 2023).

### 6.1 Exercise promoted protection increases autophagy in cardiac disease with inadequate autophagy

Numerous studies underscore the significance of autophagy in various physiological processes, including exercise adaptation, aging, and disease progression. For instance, Cobb et al. identified Idit (human FNDC5), a *Drosophila* homolog of the Irisin precursor FNDC5, as pivotal in exercise-induced improvements in cardiac autophagy. Their findings highlight the evolutionary conservation of autophagy regulation across species, influencing exercise physiology and metabolic adaptations (Cobb et al., 2023; Zhou et al., 2019). Autophagy has also been associated with enhanced clearance of protein aggregates in aging and disease contexts (Jumper et al., 2021), indicating its role in mitigating metabolic and degenerative disorders (Yu et al., 2019). Moreover, interventions that enhance autophagy, such as AMPK activation and SGLT2 (fly SLC5A11) inhibitors, have shown promise in metabolic disorders like diabetic heart and kidney conditions in mammals (Safaie et al., 2024). The *Drosophila* homolog of the mammalian SGLT2 gene, SLC5A11, plays a crucial role in central nervous system (CNS) glial cells and EB R4 neurons in flies (Park et al., 2016; Yildirim et al., 2022). It is reasonable to hypothesize that this gene may also have an important physiological function in the *Drosophila* heart tube and in the context of aging.

Altered autophagy dynamics have been implicated in skeletal muscle diseases and cardiac dysfunction in heart failure (Zirin et al., 2015). Exercise has been shown to modulate autophagy through mechanisms involving Sestrin activation, which inhibits mTORC1 and enhances mTORC2 activity, thereby coordinating metabolic processes and promoting health span (Cobb et al., 2021; Sujkowski and Wessells, 2021). Notably, high-intensity exercise has been linked to increased autophagy in the heart, underscoring the intricate interplay between autophagy and oxidative stress in diseases such as Parkinson’s disease (Zang et al., 2020a; Kong et al., 2020). In *Drosophila*, chronic exercise results in increased mitophagy in cardiac muscle mitochondria, contributing to reduced oxidative damage accumulation (Sujkowski et al., 2020). Conversely, in *Drosophila* mutants with elevated intramyocellular lipid stores, exercise training reverses lipid accumulation and concomitantly decreases autophagy, suggesting a transient regulation of autophagy in response to exercise (Sujkowski and Wessells, 2021).

Selective breeding and endurance training have been shown to improve endurance levels, cardiac performance, and autophagy in adipose tissue. The methuselah-like (mthl) gene family, downregulated by these interventions, plays a role in mediating these physiological adaptations (Sujkowski et al., 2015). Furthermore, exercise and octopamine have been demonstrated to enhance autophagy and lipolysis in various flying insects, with exercise increasing autophagy in the fat body of wild-type male *Drosophila* (Sujkowski et al., 2020). The expression of Octβ2R (β-adrenergic octopamine receptor; human HTR4) in skeletal muscle is crucial for enhancing endurance, speed, cardiac function, and fat body autophagy, indicating systemic effects across tissues (Sujkowski et al., 2020). Sestrins, evolutionarily conserved exercise-inducible proteins, are critical mediators of exercise benefits in both flies and mammals. Their knockout prevents exercise-induced adaptations in endurance and flight in *Drosophila* and impairs metabolic improvements in exercising mice, underscoring their role in diverse physiological adaptations conferred by chronic exercise (Sujkowski and Wessells, 2021).

The gene Atg2, essential for autophagy formation, is implicated in mitigating oxidative stress and delaying muscle aging during exercise. However, the precise interplay between exercise and Atg2 in muscle aging remains to be fully elucidated (Wen et al., 2023). Irisin/FNDC5, essential for multiple exercise-related benefits such as adipose tissue browning and cognitive enhancement, also plays a conserved role in upregulating autophagy, as observed between mammalian FNDC5 (Zhou et al., 2019) and its *Drosophila* homolog Idit. Notably, mutations in Idit do not directly impair mobility but specifically reduce endurance in *Drosophila* endurance models (Sujkowski and Wessells, 2021).

### 6.2 Exercise inhibits excessive autophagy in cardiac pathologies

As an effective model for studying aging, fat metabolism, adult cardiac function, and the effects of endurance exercise (Piazza and Wessells, 2011), *Drosophila* can also perform as an active model to investigate exercise and its autophagy mechanism related to cardiac function, which can compare to mammal models (Feng et al., 2019). The research on flies unveiled a nutrition-linked autophagy that responding to endurance training with improved cardiovascular function and stress resistance at advanced ages (Sujkowski et al., 2012).

Sujkowski et al. revealed that the regulation of nutrient allocation and utilization plays a critical role in modulating the onset and progression of age-related declines in cardiac function. Using mutants of dFatp (*Drosophila* Fatp; human SLC27A1), their data exhibit increased lifespan and stress resistance, altered feeding behavior, enhanced fat storage, and improved mobility, alongside impaired cardiac function. Endurance exercise effectively reverses the increased lipid storage and detrimental cardiac effects associated with dFatp mutation, albeit not fully restoring lipid levels to wild-type levels (Sujkowski et al., 2012). Autophagy and lipid metabolism cooperate closely in modulating longevity pathways, evidenced by increased lysotracker staining in the fat body of yw males following exercise training. However, yw; dFatp mutants exhibit minimal lysotracker staining irrespective of exercise, indicating a role for dFatp in autophagy regulation and cardiac performance (Sujkowski et al., 2012).

More researches employed mammal models to investigate autophagy related mechanism of cardiac function (Feng et al., 2019). For instance, Liang et al. identified lncRNA 2810403D21Rik/Mirf (*myocardial infarction-regulatory factor*) as a regulator of macroautophagy/autophagy through modulation of microRNA 26a, highlighting its role in cardiac injury and function (Liang et al., 2020). Moreover, Wang et al. discussed how exercise training restores normal autophagy function, attenuating cardiovascular disease progression associated with dysfunctional autophagy (Wang et al., 2020). Their review emphasizes the importance of autophagy and its signaling pathways in mediating exercise-induced cardiovascular benefits. Because of fundamental role of autophagy in maintaining cellular health and function, these studies underscore its relevance to exercise performance, aging, and disease progression. Comprehensive research is needed to further unravel the specific mechanisms governing autophagic regulation on exercise-increased improvement of cardiac function in cardiac disease. Using *Drosophila* model, more comprehensive information could can be made for providing understanding on the inadequate and excessive autophagy, which could potentially generate therapeutic targeting approach in various pathological conditions.

## 7 Cold stress affects *Drosophila* autophagy associated with cardiac function

Within the heart, cardiomyocytes remove dysfunctional mitochondria and other cargo using dedicated membranous particles driven by the autophagy machinery, which is enhanced during periods of cardiac stress (Nicolás-Ávila et al., 2020). Zhu et al. investigated the cardiophysiological responses of *Drosophila* larvae to acute and chronic cold stress, focusing on neuromodulators’ role in modulating cardiac function under cold conditions. Cold tolerance, crucial for ectothermic organisms, correlates closely with cardiac performance. Their research reveals that cold exposure significantly alters heart rate (HR); acute cold stress induces a marked decrease in HR, whereas chronic cold conditioning partially mitigates this effect, suggesting an acclimation process. High-performance liquid chromatography (HPLC) analysis shows reduced circulating levels of octopamine (OA) and serotonin (5-HT) in chronically cold-conditioned larvae, which likely contributes to maintaining heart function (Zhu et al., 2016; Bhide et al., 2024).

The expression of the Idit gene is intricately linked to autophagy, critical for cellular homeostasis and stress adaptation. Specifically, Idit gene expression is necessary for inducing excessive autophagy through the Atg1/Atg13 protein kinase complex, a pivotal regulator in autophagic flux. In cardiac tissue, Idit knockout significantly decreases the autophagic marker Atg8, indicating impaired autophagic machinery in the heart. Cold tolerance, essential for surviving low temperatures, relies heavily on Idit gene expression. *Drosophila* lacking Idit exhibit reduced cold tolerance, underscoring its role in thermotolerance mechanisms. Restoring Idit expression through transgenic methods restores normal cold tolerance, emphasizing its importance in cold acclimation. Moreover, Idit gene expression positively influences cardiac function; Idit knockout flies demonstrate cardiac performance equivalent to unexercised wild-type flies under stress, indicating reduced ability to cope with physiological demands (Cobb et al., 2023). Conversely, Idit overexpression enhances cardiac resilience and performance comparable to or exceeding that of exercise-trained wild-type flies, highlighting its role in cardiac adaptation.

Autophagy plays a crucial role in maintaining cardiovascular function during physical interferences and cardiac stress including exercise and cold stress in both mammal and *Drosophila* models. Dysregulation of autophagy and mitophagy contributes to myocardial contractile irregularities induced by cold stress, although specific interventions can rescue these effects. Further investigation is warranted to fully elucidate the mechanisms through which cold stress impacts cardiac function via autophagy in *Drosophila*.

## 8 Conclusion and prospects

The interplay between exercise, cold stress, and autophagy in the cardiac function of *Drosophila* has been a subject of significant research interest due to its potential implications for understanding heart health and disease mechanisms in mammals, humans, and higher organisms. Over half of the 12 *Drosophila Atg* genes are involved in autophagic cardiophysiology in fruit flies. The axis of Atg2-AMPK/Sirt1/PGC-1α (human ATG2-AMPK/SIRT1/PGC-1α) pathways mitigates age-related functional declines in skeletal and cardiac muscle in *Drosophila*. Autophagy-mTOR signaling, along with DmSestrin, plays a critical role in the relationship between post-translational modifications and their regulation of fundamental autophagy. Increasing autophagy suppresses laminopathy-induced age-dependent cardiac dysfunction accompanied by mitochondrial dysmorphology. Furthermore, nuclear remodeling and myogenic transcription factors are involved in cardiac remodeling in mice, fruit flies, and non-human primates. Exercise modulates autophagy in a context-dependent manner, improving cardiac function in *Drosophila*. Specifically, in conditions where autophagy is inadequate, exercise increases autophagic activity, thereby ameliorating cardiac disease (Cobb et al., 2023; Sujkowski et al., 2015; Sujkowski and Wessells, 2021; Sujkowski et al., 2020; Weeks et al., 2021; Wang et al., 2023; Bonilla et al., 2023). Conversely, in scenarios with excessive autophagy, exercise inhibits autophagy to protect against cardiac pathologies (Wang et al., 2020; Sujkowski et al., 2012; Liu C. Y. et al., 2018). Cold stress, a well-known environmental stressor, is also implicated in the regulation of autophagy and cardiac function in mammals and *Drosophila*.

**FIGURE 3:**
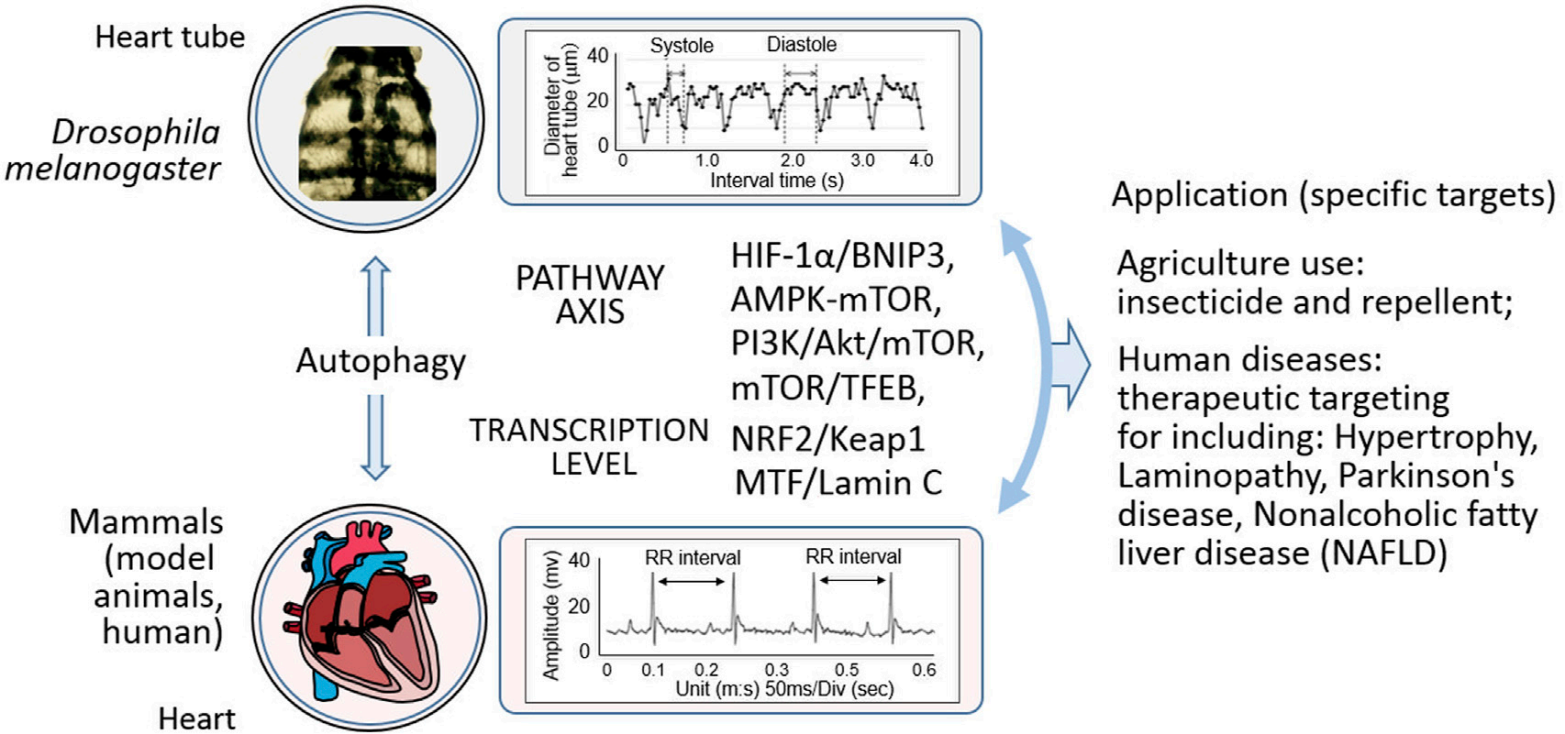
Mechanistic insights and multifaceted applications in *Drosophila* and mammals. This illustration depicts the intricate relationship wherein autophagy, and operates through diverse pathways in both *Drosophila* and mammals. It showcases how these autophagic processes impact specific targets, and further elaborates on their extensive applications. In the agricultural realm, they potentially underpin the development of novel pesticides and other relevant agents. In the medical field, they hold promise for the treatment of a spectrum of human diseases, such as myocardial hypertrophy, laminopathy, Parkinson’s disease, and non-alcoholic fatty liver disease.

The role of autophagy in cardiac hypertrophy, a condition characterized by the enlargement of the heart muscle, is complex (Li et al., 2023). In *Drosophila*, cardiac hypertrophy is influenced by Atgs, suggesting that autophagy plays a critical role in maintaining cardiac structure and function. Furthermore, the activation of autophagy suppresses cardiac laminopathy, a disease caused by mutations in the genes encoding nuclear lamins (Shaw et al., 2022; Kirkland et al., 2023). This suppression is observed in age-dependent cardiac dysfunction induced by laminopathy, with increased autophagy leading to improved cardiac function. The mechanistic link between autophagy and laminopathy involves the regulation of mitochondrial morphology and the conservation of nuclear remodeling and myogenic transcription factor integrity across species, including mice and nonhuman primates. For insights into the genetic study of heart aging in *Drosophila*, Cannon et al. provide a comprehensive analysis of cardiac-specific gene expression in aging fruit flies, drawing parallels with mammalian models (Cannon et al., 2017). The multiple signaling pathways that govern the regulation of autophagy and cardiac function in *Drosophila* are multifaceted. Atgs are central to the regulation of autophagy, and their manipulation can profoundly affect heart function (Xu et al., 2019). Additionally, the mTOR signaling pathway, a key regulator of autophagy, is associated with autophagic processes within *Drosophila*. A recent review by Griffey and Yamamoto discusses the role of macroautophagy in CNS health and disease, highlighting the conserved nature of the process and its emerging selective pathways (Griffey and Yamamoto, 2022). These findings underscore the dynamic role of autophagy in the heart’s response to various physiological and environmental stressors. Insights gained from *Drosophila* models provide a valuable foundation for further research into the molecular mechanisms underlying cardiac function and disease, with potential applications in developing therapeutic strategies for human heart conditions (Figure 3).

Recently, Shin and Worman summarized the molecular pathology of laminopathies, unveiling mechanisms of cardiomyopathic disease that are tightly related to emerin and lamin, which are critical for the nuclear envelope (Shin and Worman, 2022). As an important cellular skeletal component, nuclear lamin-associated laminopathies include progeroid disorders, striated muscle disease, adipose tissue disease, and peripheral neuropathy. The major characteristics involve the reciprocal regulation of cellular mechanics and metabolism, which are closely associated with the cellular ERK1/2 and AKT/mTOR (fly Akt/mTOR) signaling pathways (Shin and Worman, 2022). Current studies on cellular mechanics in cardiomyocytes (Santinho et al., 2024; Evers et al., 2021; Meizlish et al., 2024) should promote comprehensive research focusing on responses to mechanical stress in *Drosophila* to ultimately understand the molecular mechanisms underlying autophagic cardiopathology in both mammals and *Drosophila*.

## Glossary

2810403D21Rik/Mirf: myocardial infarction-regulatory factor
5-HT: Serotonin, also known as 5-hydroxytryptamine
AdD: adenylation domain
AKG: alpha-ketoglutarate
AMPK: adenosine 5‘-monophosphate (AMP)-activated protein kinase
Atg: autophagy-related
atl: atlastin
Atg11_C: autophagy-related protein 11, C-terminal
Atg2/VPS13_C: Atg2/VPS13, C-terminal domain
Autophagy_act_C: autophagocytosis associated protein, active-site domain
Autophagy-rel_prot_9: autophagy-related protein nine family
BARA: β-α repeated, autophagy-specific domain
BNIP3: BCL2 interacting protein 3
C2_PI3K_class_III: C2 phosphatidylinositol 3-kinase-type domain
CC: coiled-coil
cDNA: complementary DNA
Ce: *Caenorhabditis elegans*
Chorein_N: vacuolar protein sorting-associated protein 13-like, N-terminal domain
CncC: cap-and-collar C
CNS: central nervous system
COUP-TFII: chicken ovalbumin upstream promoter-factor II
CtsB1: Cathepsin B1
daw: TGFβ-INHB/activin-like ligand
DmFatp: *Drosophila* fatty acid transport protein
Dm: *Drosophila melanogaster*
DmSesn: DmSestrin
ENDOG: endonuclease G
ESCORT-III: endosomal sorting complex required for transport III
FNDC5: fibronectin type III domain-containing 5
FOXO1: forkhead box protein O1
FoxP3: forkhead/winged helix transcriptional factor P3
ER: endoplasmic reticulum
GATOR: GAP toward Rags
HBR: α-helical bundle region domain
HEK293: human embryonic kidney 293
HIF-1α: transcription factor hypoxia inducible factor-1α
HORMA: Hop1p, Rev7p and MAD2
HPLC: High-performance liquid chromatography
HR: heart rate
Hs: *Homo sapiens*
HSPs: hereditary spastic paraplegias
Keap1: kelch like ECH associated protein 1
Idit: Iditarod
Kinase: protein kinase domain
LamC: the mammalian Lamin A/C homologue
lipophagy: macroautophagy
LIR: LC3 Interacting Region motif
lncRNA: long noncoding RNA
LMNA: lamin A/C
MEF: mouse embryonic fibroblast
MTF: myogenic transcription factor
MIT: tandem microtubule interacting and transport
MS: mass spectrometry
mthl: methuselah-like
mTOR: mechanistic or mammalian target of rapamycin
mTORC1: mechanistic target of rapamycin complex 1
mTORC2: mechanistic target of rapamycin complex 2
NAFLD: non-alcoholic fatty-liver disease
NRBF2: nuclear receptor binding factor 2
Nrf2: nuclear factor erythroid 2-related factor 2
NTD: N-terminal domain
OA: octopamine
Octβ2R: β-adrenergic octopamine receptor
Peptidase C54: peptidase C54, catalytic domain
PGC-1α: peroxisome proliferator-activated receptor-gamma coactivator (PGC)-1alpha
PI3Ka: phosphoinositide 3-kinase, accessory (PIK) domain
PI3KC3: phosphatidylinositol 3-kinase catalytic subunit type 3
PI3Kc_III: phosphatidylinositol 3-/4-kinase, catalytic domain
PROPPIN: β-propellers that bind polyphosphoinositides
Rag: Ras-related GTP-binding proteins
Ras: renin-angiotensin system
RTK: receptor tyrosine kinase
SESN2: Sestrin2
Sestrin: PA26 p53-induced protein family
SETD2: SET domain containing 2, histone lysine methyltransferase
SGLT2: Sodium-Glucose Transport Protein 2
Sirt1: Sirtuin 1
Small GTPase: small GTPase
Small_GTP-bd: small GTP-binding domain
SNAREs: soluble N-ethylmaleimide-sensitive factor attachment protein receptors
SQSTM1: sequestosome 1
STX17: Syntaxin 17
TAP: tandem affinity purification
TFEB: the transcription factor EB
TGFβ: transformation growth factor beta
ThiF: THIF-type NAD/FAD binding fold domain
TRα1PV: THRA-PV, thyroid nuclear receptor alpha gene
t_SNARE: target SNARE coiled-coil homology domain
Ubl: Ubiquitin-like domain
UblA: N-terminal ubiquitin-like domain
ULK: Unc-51-like kinase
VPS15-like_hel: VPS15-like, helical domain
VPS: vacuolar protein sorting
WD40: β-propeller domain
WIPI2: WD Repeat Domain, Phosphoinositide Interacting 2
*yw*: *y* ^*1*^ *w* ^*67c23*^ fly
