# Supplementary material for: Molecular mechanism on autophagy associated cardiovascular dysfunction in Drosophila melanogaster
Source: Front Cell Dev Biol. 2025 Mar 3;13:1512341. doi: 10.3389/fcell.2025.1512341 (PMC11911378; doi:10.3389/fcell.2025.1512341)
Supplement: Supplementary file 1 [file DataSheet1.pdf]

Table S1 Homologous genes in *Drosophila*, human and mouse

Drosophila gene symbol	Human homologue	Mouse homologue	References	Links
Atg1	ULK1	Ulk1	Braden and Neufeld, 2016; Tyra et al., 2020; Zhang et al., 2023; Zhao et al., 2023	https://flybase.org/reports/FBgn0260945.htm
Atg2	ATG2B	Atg2a	Rodriguez-Fernandez and Dell'Angelica, 2015; Nagy et al., 2014; Pän et al., 2017; Xu et al., 2019; Laczkő-Dobos et al., 2021; Qin et al., 2023; Wang et al., 2023	https://flybase.org/reports/FBgn0044452.htm
Atg3	ATG3	Atg3	Chang et al., 2013; Soukup et al., 2016; Yue et al., 2022	https://flybase.org/reports/FBgn0031298.htm
Atg4a	ATG4A	Atg4a	Del Caño-Espinel et al., 2015; Billes et al., 2018; Cunningham et al., 2020	https://flybase.org/reports/FBgn0031298.htm
Atg4b	ATG4D	Atg4d	Thumm and Kadowaki,2001; Cunningham et al., 2020; Li et al., 2023	https://flybase.org/reports/FBgn0038325.htm
Atg5	ATG5	Atg5	Kim et al., 2016; Lin et al., 2020; Bjedov et al., 2020; Zhu et al., 2021; Krzystek et al., 2023; Nan et al., 2024	https://flybase.org/reports/FBgn0029943.htm
Atg6	BECN1	Becn1	Shravage et al., 2013; M'Angale and Staveley, 2016; Na et al., 2018; Zhao et al., 2018; Shen et al., 2022	https://flybase.org/reports/FBgn0264325.htm
Atg7	ATG7	Atg7	Yan et al., 2019; Han et al., 2020; Cai et al., 2020; Donde et al., 2020; Zhu et al., 2021	https://flybase.org/reports/FBgn0034366.htm
Atg8a, Atg8b	GABARAP	Gabarap	Nilangekar et al., 2019; Jacomin et al., 2020; Jipa et al., 2021; Rahman et al., 2022; Tsapras and Nezis, 2022; Rogov et al., 2023; Xu et al., 2024; Hatfield and Johnson, 2024	https://flybase.org/reports/FBgn0052672.htm https://flybase.org/reports/FBgn0038539.htm
Atg9	ATG9A	Atg9b	Xu et al., 2019; Kiss et al., 2020; Li et al., 2023; Yi et al., 2024	https://flybase.org/reports/FBgn0034110.htm
Atg10	ATG10	Atg10	Al L Abaquita et al. 2021 Damulewicz et al. 2022 Abaquita et al. 2023 Demir and Kacew 2023 Perlegos et al. 2024 Umargamwala et al. 2024	https://flybase.org/reports/FBgn0040780.htm
Atg12	ATG12	Atg12	Lin et al., 2020; Murakawa et al., 2020; Zhu et al., 2021; Liu et al., 2023; Nan et al., 2024	https://flybase.org/reports/FBgn0036255.htm
Atg13	ATG13	Atg13	Nezis et al., 2010; Nagy et al., 2014; Tyra et al., 2020	https://flybase.org/reports/FBgn0261108.htm
Atg14	ATG14	Atg14	Hegedűs et al., 2016; Melani et al., 2017; Takáts et al., 2021; Xu et al., 2022	https://flybase.org/reports/FBgn0039636.htm
Atg16	ATG16L1	Atg16l1	Varga et al., 2016; Nagy et al., 2017; Szabó et al., 2023	https://flybase.org/reports/FBgn0039705.htm
Atg17	RB1CC1	Rb1cc1	Bánrőti et al., 2012; Kim et al., 2013; Nagy et al., 2014; Cao et al., 2017; Melani et al., 2017; Yang et al., 2020	https://flybase.org/reports/FBgn0037363.htm

Atg18a, Atg18b	WIPI2	Wipi2	Spradling et al., 1999; Nagy et al., 2014; Xu et al., 2019; Murakawa et al., 2020; Cunningham et al., 2020; Yang et al., 2020; Murakawa et al., 2022; Szabó et al., 2023	https://flybase.org/reports/FBgn0035850.htm https://flybase.org/reports/FBgn0032935.htm
Atg101	ATG101	Atg101	Bánrőti et al., 2012; Hegedűs et al., 2014; Nagy et al., 2014; Guo et al., 2019; Zhu et al., 2021; Yue et al., 2022	https://flybase.org/reports/FBgn0030960.htm
Uvrag	UVRAG	Uvrag	Csizmadia et al., 2018; Takáts et al., 2021; Manzöger et al., 2021; Xu et al., 2022; Szabó et al., 2023	https://flybase.org/reports/FBgn0032499.htm
Vps15	VPS15	Vps15	Wu et al., 2007; Lindmo et al., 2008; Abe et al., 2009; Anding and Baehrecke,2015; Melani et al., 2017	https://flybase.org/reports/FBgn0260935.htm
Vps34	Vps34	Vps34	Melani et al., 2017; Csizmadia et al., 2018; Valko et al., 2022; Szabó et al., 2023	https://flybase.org/reports/FBgn0015277.htm
Aduk	ULK3	Ulk3	Park et al., 2013; Braden and Neufeld, 2016	https://flybase.org/reports/FBgn0037679.htm
Rab2	RAB2A	Rab2a	Lőrincz et al., 2017; Mallik et al., 2017; Fujita et al., 2017; Lund et al., 2018; Lund et al., 2021; Götz et al., 2021	https://flybase.org/reports/FBgn0014009.htm
Rab7	RAB7A	Rab7a	Cherry et al., 2013; Janssens et al., 2014; Hegedűs et al., 2016; Basargekar et al., 2020; Dehnen et al., 2020; Patel et al., 2020; Krzystek et al., 2023; Vöing et al., 2023	https://flybase.org/reports/FBgn0015795.htm
Syx17	STX17	Stx17	Zhang et al., 2019; Rong et al., 2022; Xu et al., 2022; Liu et al., 2023; Krzystek et al., 2023	https://flybase.org/reports/FBgn0035540.htm
Arl8	ARL8A	Arl8a	Jha et al., 2016; Rosa-Ferreira et al., 2018; Vukoja et al., 2018; Boda et al., 2019; Lund et al., 2021; Kanca et al., 2022	https://flybase.org/reports/FBgn0037551.htm
Sesn	SESN1, SESN2, SESN3	Sesn1, Sesn2, Sesn3	Lee et al., 2010; Lee et al., 2012; Singh and Chowdhuri,2018; Gu et al., 2022; Sujkowski et al., 2022	https://flybase.org/reports/FBgn0034897.htm
dTOR	MTOR	Mtor	Xi et al., 2019; Zeng et al., 2020; Rambur et al., 2020; Devilliers et al., 2021; Zhou et al., 2021; Spitz et al., 2022; Sanal et al., 2023; Srivastav et al., 2024	https://flybase.org/reports/FBgn0021796.htm
4E-BP	4E-BP1, 4E-BP2, 4E-BP3	4e-bp1, 4e-bp2, 4e-bp3	Feuillette et al., 2020; Vandehoef et al., 2020; Jin et al., 2020; Myers et al., 2022; Mushtaq et al., 2022; Santalla et al., 2022; Bahuguna et al., 2022; Kim et al., 2023; Garcia et al., 2024	https://flybase.org/reports/FBgn0261560.htm
Set2	SETD2	Setd2	Mitra et al., 2021; McCarthy et al., 2022; Lindehell et al.,	https://flybase.org/reports/FBgn0030486.htm

			2023; Mitra et al., 2024; Brockett et al., 2024	
dFatp	FATP	Fatp	Van Den Brink et al., 2018; Yan et al., 2019; Pandey et al., 2021; Girard et al., 2021; Guichard et al., 2023; Le et al., 2024	https://flybase.org/reports/FBgn0267828.htm
TGFβ	MAP3K7	Map3k7	Takatsu et al., 2000; Mihaly et al., 2001; Yang and Su,2011; Paquette et al., 2012; Stronach et al., 2014; West et al., 2015; Chen et al., 2017; Barros and Bossing,2021; Das et al., 2023; Hu et al., 2024	https://flybase.org/reports/FBgn0026323.htm
CtsB	CTSB	Ctsb	Kuronen et al., 2009; Tseng et al., 2013; Kanca et al., 2022	https://flybase.org/reports/FBgn0030521.htm
ref (2) P	SQSTM1	Sqstm1	de Castro et al., 2013; DeVorkin and Gorski,2014; Jain et al., 2015; Carroll et al., 2018; Yan et al., 2019; Hurley and Staveley,2021; Huang et al., 2022; Bhattacharjee et al., 2022; Tan et al., 2024	https://flybase.org/reports/FBgn0003231.htm
LamC	LMNB1, LMNB2, LMNA	Lmnb1, Lmnb2, Lmna	Dialynas et al., 2012; Zwerger et al., 2013; Dialynas et al., 2015; Li et al., 2016; Bhide et al., 2018; Chandran et al., 2019; Hinz et al., 2021; Shaw et al., 2022; Walker et al., 2023	https://flybase.org/reports/FBgn0010397.htm
Sirt1	SIRT1	Sirt1	Banerjee et al., 2012; Hong et al., 2012; Adhikari et al., 2019; Jacomin et al., 2020; Damschroder et al., 2022; Miao et al., 2022; Palu et al., 2022; Hao et al., 2023; Larnerd et al., 2023; Willnow and Teleman,2024; Lee and Min,2024	https://flybase.org/reports/FBgn0024291.htm
Idit	FNDC5	Fndc5	Cobb et al., 2023	https://flybase.org/reports/FBgn0053143.htm
Nrf2	NRF2	Nrf2	Castillo-Quan et al., 2016; Bhide et al., 2018; Spiers et al., 2019; Saeedi et al., 2020; Chew et al., 2021; Tsakiri et al., 2021; Bhattacharjee et al., 2022; Carlson et al., 2022; Na et al., 2022; Neidviecky and Deng,2023; Tan et al., 2024; Tsuji et al., 2024; Xi et al., 2024; Wen et al., 2024	https://flybase.org/reports/FBgn0262975.htm
eIF4E	EIF4E	Eif4e	Wilhelm et al., 2003; Zappavigna et al., 2004; Parra-Palau et al., 2005; Titlow et al., 2020; Buddika et al., 2020; Santalla et al., 2022; Layana et al., 2023	https://flybase.org/reports/FBgn0015218.htm

Akt	AKT1, AKT2, AKT3	Akt1, Akt2, Akt3	Roth et al., 2018; Kang et al., 2018; Borreguero-Muñoz et al., 2019; Santabárbara-Ruiz et al., 2019; Hwang et al., 2019; Sharrock et al., 2019; Chen et al., 2019; Shohayeb et al., 2020; Sun et al., 2020; Kierdorf et al., 2020; Wang et al., 2020; Cheng et al., 2021; Cheng et al., 2022; Kim,2022; Kanaoka et al., 2023; Na et al., 2023	https://flybase.org/reports/FBgn0010379.htm
AMPK	AMPK	Prkaa2	Su et al., 2019; Yuan et al., 2020; Chandran et al., 2019; Liu et al., 2021; Marzano et al., 2021; Lin et al., 2022; Yuh Chew et al., 2022; Livelo et al., 2023; Borkowsky et al., 2023; Guo et al., 2023; Zhao et al., 2023; Li et al., 2024	https://flybase.org/reports/FBgn0023169.htm
SLC5A11	SGLT2	Sglt2	Dus et al., 2013; Park et al., 2016; Ugrankar et al., 2018	https://flybase.org/reports/FBgn0031998.htm
Ras	HRAS	Hras	Ito and Igaki,2021; Enomoto et al., 2021; Rackley et al., 2021; Dong et al., 2021; Kong et al., 2021; Singh et al., 2023; Zhang et al., 2024; Karunaraj et al., 2024	https://flybase.org/reports/FBgn0003205.htm
Raf	BRAF	Braf	Mark et al., 1987; Sun et al., 2020; Xie et al., 2023	https://flybase.org/reports/FBgn0003079.htm
Octβ2R	HTR4	Htr4	McKinney et al., 2020; Zhao et al., 2021; Wong et al., 2021; Deshpande et al., 2022; Bonanno et al., 2024; Lv et al., 2024	https://flybase.org/reports/FBgn0038063.htm
EndoG	ENDOG	Endog	Loll et al., 2009; Seo et al., 2014; Yu et al., 2017; Wang et al., 2021	https://flybase.org/reports/FBgn0289744.htm
Raptor	RPTOR	Rptor	Sarbassov et al., 2004; Lee and Chung,2007; Li et al., 2019; Jevitt et al., 2021; Formica et al., 2021	https://flybase.org/reports/FBgn0029840.htm
Rictor	RICTOR	Rictor	Sarbassov et al., 2004; Sarbassov et al., 2005; Lee and Chung,2007; Taslim et al., 2023	https://flybase.org/reports/FBgn0031006.htm
daw	INHBA	Inhba	Langerak et al., 2018; Wu et al., 2022; Mallick et al., 2024	https://flybase.org/reports/FBgn0031461.htm
ESCRT-III	CHMP4B	Chmp4b	McMillan et al., 2017; Arii et al., 2018; Capalbo et al., 2019; Warecki et al., 2020; Dubey et al., 2022; Baeumers et al., 2022; Mathieu et al., 2022; Marie et al., 2023; Bruelle et al., 2023; Esmangart de Bournonville et al., 2024; Hermant et al., 2024	https://flybase.org/reports/FBgn0086656.htm
PGC-α	PPARGC1	Ppargc1a	Wei et al., 2018; Basar et al., 2019; George and Jacobs,2019; George and Jacobs,2019; Roy et al., 2023; Weisz et al., 2024	https://flybase.org/reports/FBgn0037248.htm

Vps4	VPS4A, VPS4B	Vps4a, Vps4b	Rodahl et al., 2009; Legent et al., 2015; Wang et al., 2019	https://flybase.org/reports/FBgn0283469.htm
FOXO	FOXO3	Foxo3	Poe et al., 2020; Wagner et al., 2021; Martin et al., 2021; Zhao et al., 2021; Guo et al., 2022; Martínez Corrales et al., 2022; Nam et al., 2022; Chen et al., 2022; Frendo-Cumbo et al., 2022; Jin et al., 2023; Dark et al., 2024; Kosakamoto et al., 2024	https://flybase.org/reports/FBgn0038197.htm
SERCA	ATP2A1, ATP2A2, ATP2A3	Atp2a1, Atp2a2, Atp2a3	Balcazar et al., 2018; Suisse and Treisman,2019; Solana-Manrique et al., 2021; Zhu et al., 2022; Santalla et al., 2022; Schiemann et al., 2022; Weiss et al., 2022; Maurya and Tapadia,2023	https://flybase.org/reports/FBgn0263006.htm

Note: The information on homologous genes of *Drosophila*, humans and mice was retrieved from the databases of Flybase (https://flybase.org/), NCBI (https://www.ncbi.nlm.nih.gov/gene/), HGNC (https://www.genenames.org/), and MGI (https://www.informatics.jax.org/).
